# Japi 2.0, a gaming platform to stimulate cognitive and socio-emotional skills in early childhood: results of a pilot randomized controlled trial

**DOI:** 10.1186/s13034-026-01133-1

**Published:** 2026-07-21

**Authors:** Jorge Gaete, Natalia Ríos, Cristian A. Rojas-Barahona, Gabriel Salgado, Saray Ramírez, Ricardo Araya

**Affiliations:** 1 Research Center for Student Mental Health (ISME), School of Education, Faculty of Social Sciences, Universidad de los Andes, Santiago, Chile; 2 Center for the Well-Being and Development of Adolescents and Children in the Digital Age (BAND), Santiago, Chile; 3 Faculty of Psychology, Research Center on Cognitive Sciences, Universidad de Talca, Talca, Chile; 4 Millennium Science Initiative Program, ANID, Millenium Nucleus for the Sciences of Learning, Talca, Chile; 5 School of Nursing, Faculty of Nursing and Obstetrics, Universidad de los Andes, Santiago, Chile; 6 Instituto Nacional del Tórax, Servicio de Salud Metropolitano Oriente, Santiago, Chile; 7 Health Service and Population Research Department, Institute of Psychiatry, Psychology and Neuroscience, King’s College London, London, UK

## Abstract

**Background:**

Early childhood is a sensitive developmental period for the acquisition of cognitive and socio-emotional skills that are strongly associated with later mental health, academic achievement, and social functioning. Children growing up in socioeconomically disadvantaged contexts are at increased risk for early emotional and behavioral difficulties, highlighting the need for accessible, scalable, and developmentally appropriate preventive interventions. Japi 2.0 is a tablet-based gaming platform designed to stimulate six interrelated cognitive and socio-emotional skills—working memory, inhibitory control, emotion recognition, social competence, empathy, and cognitive planning—through structured play in preschool settings.

**Methods:**

We conducted a pilot cluster randomized controlled trial in low-income preschools in Santiago, Chile. Schools were randomized to either the Japi 2.0 intervention or treatment as usual. The intervention consisted of 24 individual sessions delivered over 12 weeks during regular class hours. Primary outcomes were acceptability (perceived by children, early years educators, and parents) and feasibility (recruitment, retention, attendance, intervention delivery, and data completeness). Secondary outcomes explored preliminary changes in cognitive, socio-emotional, and behavioral functioning using standardized assessments and parent- and teacher-reported measures. Exploratory analyses were conducted using multilevel models adjusted for baseline scores and clustering, based on available outcome data from randomized participants.

**Results:**

Japi 2.0 showed high acceptability across stakeholders. Educators and parents reported that the game and the accompanying web-based dashboard were engaging, age-appropriate, easy to use, and pedagogically relevant. Children reported high levels of enjoyment and positive emotional experiences during gameplay. Feasibility indicators were satisfactory: most planned sessions were delivered as scheduled, attendance was high, and outcome data completeness was adequate. Exploratory analyses indicated improvements over time in both groups across most outcomes. Preliminary exploratory signals favoring the intervention were observed for empathy and parent-reported emotional and behavioral difficulties; however, these findings should be interpreted cautiously given the small number of clusters and exploratory nature of the analyses.

**Conclusions:**

This pilot trial demonstrates that Japi 2.0 is a feasible and highly acceptable digital intervention for preschool children in low-income educational settings. Although efficacy findings are preliminary, results support progression to a fully powered randomized controlled trial to evaluate effectiveness and longer-term impacts on cognitive, socio-emotional, and behavioral development.

*Trial registration* Clinical Trials NCT06420544, May 20th, 2024. https://www.clinicaltrials.gov/study/ NCT06420544.

**Supplementary Information:**

The online version contains supplementary material available at https://doi.org/10.1186/s13034-026-01133-1.

## Introduction

Mental health disorders are among the leading contributors to the global burden of disease, with their relative impact expected to increase worldwide [1, 2]. Importantly, mental health problems are already present early in life. Epidemiological evidence indicates that a substantial proportion of preschool children experience emotional and behavioral difficulties. A recent meta-analysis including more than 18,000 children aged 1 to 7 years reported a pooled prevalence of mental disorders of approximately 20%, with oppositional defiant disorder, attention-deficit/hyperactivity disorder, and anxiety disorders being the most common conditions [3].

Early mental health problems are not randomly distributed. Children exposed to socioeconomic adversity are at particularly high risk. Poverty, lower parental education, reduced cognitive and emotional stimulation in the home, and family dysfunction have consistently been identified as key risk factors for early emotional and behavioral problems [4, 5]. These findings highlight the importance of individual, familial, and contextual factors in explaining early mental health disparities.

From a life-course perspective, early prevention is a critical strategy. Most mental disorders have their onset in childhood or adolescence [6], highlighting early development as a critical period for intervention. However, translating this knowledge into scalable and effective preventive strategies remains a major challenge, particularly in real-world educational settings [6]. There is growing evidence that strengthening cognitive and psychosocial skills in early childhood may reduce later risk for mental health problems. For example, difficulties in emotion regulation and social problem-solving have been associated with an increased incidence of depression and other internalizing disorders [7, 8].

Early childhood represents a sensitive developmental window for the acquisition of cognitive and socio-emotional skills. Beyond traditional distinctions between cognitive and socio-emotional skills, current developmental frameworks emphasize the role of integrated self-regulatory processes—including executive functions and socio-emotional competencies—in shaping long-term outcomes [9, 10]. In early childhood, these domains are highly interrelated and jointly support adaptive functioning, learning, and social development [11, 12]. This integrated perspective informed the selection of the target skills addressed by the Japi 2.0 intervention. For instance, executive functions, including working memory, inhibitory control, and cognitive planning, are central to self-regulation and adaptive functioning. These processes are closely linked to academic performance, task persistence, social adjustment, and reduced behavioral problems [13–15]. Neurodevelopmental research shows that the brain systems supporting executive functions and emotion regulation exhibit marked plasticity during early childhood, making this period particularly suitable for targeted interventions [16, 17]. Importantly, most of the evidence linking early psychosocial skills to later outcomes originates from high-income countries, underscoring the need for replication and innovation in low- and middle-income contexts [18, 19].

### Cognitive and socio-emotional skills targeted by Japi 2.0

While early childhood represents a critical developmental window for the acquisition of cognitive and socio-emotional skills [20, 21], translating this knowledge into scalable and context-embedded interventions remains a major challenge, particularly in low-resource educational settings [22, 23]. In this context, digital game-based platforms have emerged as a promising approach to deliver structured and engaging learning experiences that can be integrated into routine classroom activities [24, 25]. Japi 2.0 was developed to operationalize these principles by combining repeated practice, progressive task difficulty, and play-based engagement to stimulate core cognitive and socio-emotional skills in early childhood. Consistent with the theoretical framework and empirical rationale described in the published protocol [26, 27], Japi 2.0 was designed to stimulate six key skills: working memory, inhibitory control, emotion recognition, social competence, empathy, and cognitive planning. These skills were selected based on their developmental relevance, malleability in early childhood, and demonstrated associations with later mental health and educational outcomes.

Working memory is a core executive function defined as the capacity to temporarily store and manipulate information to guide goal-directed behavior [28]. It shows rapid development during the preschool years, particularly between ages 3 and 5 [29]. Individual differences in working memory during early childhood predict later academic performance in reading and mathematics [30, 31], as well as vulnerability to externalizing and internalizing mental health problems [32, 33]. Experimental studies suggest that working memory is at least partially trainable in young children through structured cognitive activities, including game-based approaches, although the durability and generalization of training effects remain under investigation [34, 35].

Inhibitory control, another core executive function, refers to the ability to suppress prepotent or impulsive responses in favor of goal-relevant actions [36]. This capacity undergoes substantial development during the preschool period and is shaped by both neurobiological maturation and caregiving environments [37]. Poor inhibitory control has been associated with aggressive behavior, hyperactivity, and later risk behaviors [38], whereas very high inhibitory control in children with elevated negative emotionality may increase vulnerability to internalizing symptoms such as anxiety and social withdrawal [39, 40]. Evidence from longitudinal and intervention studies indicates that inhibitory control is malleable in early childhood and contributes independently to academic readiness and literacy skills [11, 12].

Beyond executive functions, emotion recognition is a foundational socio-emotional skill that supports social understanding and self-regulation. Preschool children show rapid improvements in the ability to identify basic emotions in facial and bodily expressions, with clear developmental gains between ages 3 and 5 [41, 42]. Deficits in emotion recognition have been linked to peer difficulties, academic problems, and increased risk of behavioral and emotional symptoms [43, 44]. Importantly, recent intervention studies demonstrate that emotion recognition is a trainable skill in preschool and kindergarten children when developmentally appropriate and interactive methods are used [45].

Social competence refers to the ability to interact effectively with others, resolve interpersonal conflicts, and navigate social situations in adaptive ways [46]. It encompasses a constellation of emotional, cognitive, and behavioral skills that develop progressively from early childhood onward. Poor social competence in preschool has been associated with peer rejection, low self-esteem, and elevated risk of later behavioral problems [47, 48]. Randomized trials of school-based interventions indicate that structured programs targeting social and emotional learning can improve social competence and reduce social withdrawal in young children [49, 50].

Empathy, defined as the capacity to understand and share the emotional states of others, is a critical component of socio-emotional development and prosocial behavior. Empathic abilities emerge early in life and continue to consolidate during the preschool years, as children become increasingly able to differentiate their own emotions from those of others [51]. Higher levels of empathy in early childhood are associated with prosocial behavior, better peer relationships, and lower levels of aggressive conduct [52]. Empathy has also been identified as a modifiable skill: intervention studies using emotion-focused narratives, perspective-taking tasks, and structured social scenarios have demonstrated improvements in empathic understanding among preschool-aged children [50, 53].

Finally, cognitive planning refers to the ability to anticipate future actions, organize steps toward a goal, and flexibly adjust strategies when conditions change. Planning is considered a higher-order executive function that builds upon working memory and inhibitory control and shows marked development during early childhood [54, 55]. Deficits in planning have been associated with difficulties in academic tasks, problem-solving, and adaptive functioning. Although less frequently targeted in preschool interventions, emerging evidence suggests that planning skills can be stimulated through structured, play-based tasks that require sequencing, foresight, and goal management [54, 56].

Taken together, these six skills represent interrelated but distinct developmental capacities that jointly support self-regulation, social adaptation, and mental health. The design of Japi 2.0 reflects this integrative framework by embedding tasks that systematically stimulate each skill within a single, age-appropriate, game-based platform, as described in the study protocol [27].

Interventions targeting cognitive and socio-emotional processes in early childhood may produce different levels of transfer. Proximal or “near transfer” effects are typically observed in tasks closely aligned with the trained skills, reflecting improvements in the specific cognitive or socio-emotional processes directly practiced during the intervention. In contrast, more distal or “far transfer” effects—such as changes in broader behavioral functioning, including emotional and behavioral difficulties or prosocial behavior—are less consistently observed and may require longer exposure, higher intervention intensity, or additional contextual supports [57, 58].

In this context, Japi 2.0 is primarily expected to influence proximal cognitive and socio-emotional skills that are directly targeted through structured gameplay. Any potential changes in broader behavioral outcomes should therefore be interpreted cautiously, particularly within the timeframe of a pilot intervention, as such outcomes may reflect more complex and distal processes that are not expected to change substantially in early-phase trials.

Building on this theoretical and empirical foundation, Japi 2.0 was developed as a tablet-based gaming platform designed to stimulate six interrelated cognitive and socio-emotional skills—working memory, inhibitory control, emotion recognition, social competence, empathy, and cognitive planning—during a sensitive period of early childhood development [27]. By integrating these skills within a single, structured, and developmentally appropriate digital environment, Japi 2.0 seeks to promote core self-regulatory and socio-emotional capacities that are relevant for mental health, learning, and social functioning, particularly among children exposed to socioeconomic adversity. Japi 2.0 is grounded in a set of complementary mechanisms that are hypothesized to support cognitive and socio-emotional skill development in early childhood. First, the intervention provides repeated and structured exposure to tasks targeting specific processes, allowing for practice-based reinforcement of emerging skills [34]. Second, activities are designed with progressive levels of difficulty, adapting task demands to children’s performance and promoting sustained cognitive engagement. Third, the use of interactive, play-based elements is intended to enhance intrinsic motivation, which is a key driver of learning in early childhood [59, 60]. Fourth, tasks are embedded in familiar and socially meaningful contexts, which may facilitate understanding and support the application of learned skills beyond the immediate task [23]. Together, these mechanisms provide a theoretically informed pathway through which structured gameplay may influence proximal cognitive and socio-emotional processes, while also supporting engagement and adherence within routine classroom settings.

Consistent with recommendations for early-phase intervention research [61, 62], the present study was conceived as a pilot randomized controlled trial with a primary focus on evaluating the acceptability and feasibility of implementing Japi 2.0 in low-income preschool settings in Chile. Accordingly, the study was not designed to establish preventive effectiveness, but rather to assess implementation processes and generate preliminary signals of change in proximal outcomes.

In line with the exploratory nature of pilot studies, we examined changes in cognitive, socio-emotional, and behavioral outcomes associated with the six targeted skills. However, given the scope and duration of the intervention, as well as the early-phase design, changes in broader behavioral outcomes were considered distal and not expected to show substantial effects. This dual focus aims to generate critical implementation and usability evidence, while providing initial signals to inform the refinement of the intervention and the design of a future, adequately powered randomized controlled trial.

While early interventions have the potential to contribute to long-term prevention, the present study focuses on short-term implementation outcomes and exploratory changes in targeted skills; therefore, any implications for prevention should be considered preliminary and hypothesis-generating.

### Aims and objectives

The primary aim of this study was to evaluate the acceptability and feasibility of implementing *Japi 2.0*, a tablet-based gaming platform designed to stimulate six cognitive and socio-emotional skills (working memory, inhibitory control, emotion recognition, social competence, empathy, and cognitive planning), in low-income preschool settings in Santiago, Chile.

#### Primary objectives

1. To assess the acceptability of Japi 2.0 among preschool children and early years educators, including perceptions of enjoyment, relevance, usability, and satisfaction with the gaming platform and its implementation in the classroom context.
2. To assess the feasibility of delivering Japi 2.0 within regular preschool routines, including recruitment and retention of schools and participants, session attendance, intervention delivery as planned, and completeness of outcome data collection.

#### Secondary (exploratory) objectives

1. To explore preliminary changes in cognitive outcomes, specifically working memory, inhibitory control, and cognitive planning, following participation in the Japi 2.0 intervention.
2. To explore preliminary changes in socio-emotional outcomes, including emotion recognition, social competence, and empathy, following participation in the intervention.
3. To explore changes in behavioral and psychological functioning, as reported by parents and educators: behavioral difficulties and prosocial behavior.

Consistent with recommendations for pilot and feasibility studies, analyses addressing secondary objectives were considered exploratory and were not intended to provide definitive evidence of effectiveness, but rather to inform refinement of the intervention, selection of outcome measures, and design parameters for a future fully powered randomized controlled trial.

## Methods

### Study design, participants, and settings

This is a pilot randomized controlled trial, parallel-group type, in which “Japi 2.0”, a gaming platform that stimulates cognitive and socio-emotional skills in early childhood, is compared to standard school curricula in control schools. Seven schools with low socio-economic status, located in Santiago, Chile, were recruited and allocated using a 1:1 stratified randomization procedure based on school size (Strata 1: schools with one class and Strata 2: schools with two classes) to ensure balanced groups.

#### Inclusion criteria for schools

1. Schools located in Santiago (Chile).
2. Schools with Preschool Education.
3. Mixed-sex schools.
4. Schools with high vulnerability (≥ 75%), as stated in the School Vulnerability Index – National System of Equality Allocation (IVE-SINAE). This index is the proportion of high-vulnerability students in a given school, considering socioeconomic variables such as the mother’s educational level, the father’s educational level, and total monthly household income, among others [26].

#### Exclusion criteria for schools

1. Three or more classes in Preschool. This criterion was considered for economic and practical reasons.
2. Implementing a manualized program to promote cognitive or social-emotional skills.
3. Participating in a similar study.

#### Inclusion criteria for students

1. Children attending pre-kindergarten (aged around 4 years old).

#### Exclusion criteria for students

1. Children with intellectual disability. Children with intellectual disability were excluded because several study assessments required developmental and cognitive abilities necessary to understand task instructions and complete standardized cognitive measures. In addition, the intervention was initially designed for implementation within mainstream preschool classroom settings among typically developing children.
2. Children unable to understand and speak Spanish. All instructions of the video game and measurements are in Spanish.

### Sample size and recruitment

This study was designed as a pilot randomized controlled trial, and therefore the sample size was not determined based on formal power calculations to detect statistically significant intervention effects. Instead, the sample size was guided by methodological recommendations for pilot studies, which emphasize the estimation of feasibility parameters, recruitment and retention rates, and variability of outcome measures to inform the design of a future definitive trial [63–65].

At the planning stage, the study aimed to recruit approximately 20 children per school, across six to seven schools, to achieve an anticipated sample of around 120 children, which was considered sufficient to evaluate acceptability, feasibility, and data completeness, and to obtain preliminary estimates of outcome variability.

The study was conducted from March 2024 to December 2024. A total of 31 eligible schools were invited to participate, of which seven schools agreed and were randomized. These schools enrolled a total of 284 eligible students prior to consent procedures. Of these, 184 students were allocated to the intervention group (four schools) and 100 students to the control group (three schools).

**Fig. 1:**
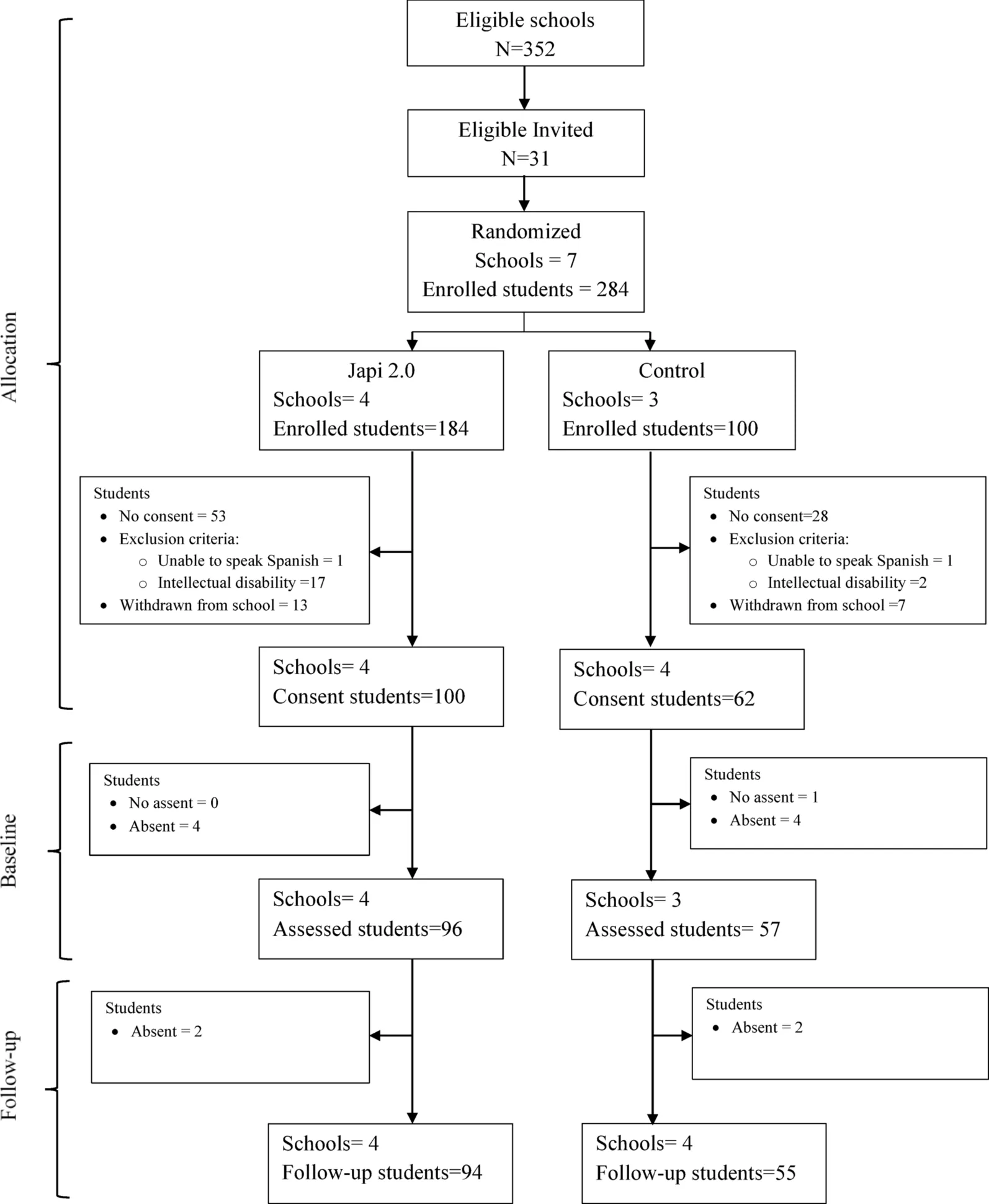
Flowchart

Following the consent process and application of exclusion criteria (including inability to speak Spanish, intellectual disability, and school withdrawal), 162 students provided parental consent (100 in the intervention group and 62 in the control group). Baseline assessments were completed for 153 students (96 in the intervention group across four schools and 57 in the control group across three schools). At post-intervention follow-up, outcome data were available for 149 students, corresponding to 94 children in the intervention group and 55 children in the control group (see Fig. 1). Several losses during recruitment and enrollment were related to administrative, logistical, or scheduling constraints at the school level, as well as incomplete consent, inability to speak Spanish, having intellectual disability, or absence during assessment procedures at the student level.

Although the achieved sample size was smaller than initially anticipated, it was considered adequate for the primary aims of the study, namely the evaluation of acceptability and feasibility, and for conducting exploratory analyses of secondary outcomes. Consistent with the pilot nature of the trial and the cluster-randomized design, the study was not powered to detect small or moderate between-group differences, and all analyses of secondary outcomes were interpreted as exploratory.

### Randomization and allocation

Randomization was conducted at the school (cluster) level after eligible schools agreed to participate and prior to the initiation of baseline assessments. In accordance with the study protocol [27], schools were randomized using a stratified randomization procedure based on school size, defined as: (i) schools with one pre-kindergarten class (small schools) and (ii) schools with two pre-kindergarten classes (large schools).

Within each stratum, schools were allocated to the intervention group (Japi 2.0) or the control group using a computer-generated random allocation sequence with a 1:1 allocation ratio. The randomization sequence was generated by a member of the research team who was not involved in recruitment, outcome assessment, or intervention delivery.

A total of seven schools were randomized: four schools were allocated to the intervention group and three schools to the control group. Allocation concealment was maintained up to the point of randomization, as schools were enrolled and consented prior to knowledge of group assignment. Once randomization was completed, schools were informed of their allocation by a research assistant.

Due to the nature of the intervention, concealment of allocation after randomization was not feasible for schools, early years educators, or research assistants involved in implementation. However, procedures for assessor blinding for secondary outcomes were implemented, as described in the Blinding section.

### Blinding

Due to the nature of the intervention, blinding of participants and early years educators was not feasible. The Japi 2.0 intervention involves active interaction with a tablet-based gaming platform delivered during regular classroom activities; therefore, children, educators, and research assistants involved in the implementation were necessarily aware of group allocation.

However, to minimize potential assessment bias, blinding was implemented at the level of outcome assessment for secondary outcomes. Specifically, evaluators responsible for administering the cognitive and socio-emotional assessments to children at baseline and post-intervention were blinded to group allocation. Evaluators were not involved in the delivery of the intervention and were instructed not to discuss intervention-related activities with school staff or students.

Primary outcomes related to acceptability and feasibility were inherently intervention-specific and therefore could not be blinded. These outcomes relied on self-report or assisted-report measures completed by early years educators, parents, and students at the end of the intervention period.

This partial blinding strategy is consistent with the study’s pilot design and with recommendations for behavioral and educational intervention trials, where full blinding is often not possible but assessor blinding can reduce the risk of detection bias [27].

### Intervention: Japi 2.0

Japi 2.0 is a tablet-based, game-based intervention designed to stimulate cognitive and socio-emotional skills in preschool children through structured, developmentally appropriate play. The intervention was delivered in the school setting during regular class hours and was implemented with the structured support of early years educators and research assistants, as described in the study protocol [27]. Early years educators and research assistants supervised sessions, provided general instructions, and facilitated engagement. Therefore, the intervention as delivered reflects a combined model integrating the digital platform with adult-mediated support during implementation.

#### Structure and delivery

The intervention consists of 24 sessions, delivered over 12 consecutive weeks, with two sessions per week, each lasting approximately 15 min. Sessions were conducted individually using tablet devices and headphones, allowing children to interact autonomously with the game while receiving standardized audio instructions. All instructions and feedback within the game were delivered through pre-recorded audio to ensure consistency across participants and sessions.

A recurring animated character (a ferret) guided children throughout the intervention, introducing each session and explaining the tasks to be completed. Sessions followed a consistent internal structure, comprising four activities per session, with each activity including multiple trials of increasing difficulty to accommodate individual differences in performance and promote skill progression.

#### Targeted skills and progression

Japi 2.0 was designed to stimulate six interrelated skills: emotion recognition, inhibitory control, working memory, social competence, empathy, and cognitive planning. The intervention follows a progressive and modular structure, in which sessions are grouped by primary skill targets while maintaining integration across domains:

- Sessions 1–8 primarily focus on emotion recognition and inhibitory control, using tasks that require children to identify emotions in faces and situations, selectively respond to target stimuli, and inhibit responses to non-target stimuli.
- Sessions 9–16 emphasize working memory and social competence, incorporating tasks that involve memorizing and reproducing sequences, ordering events, identifying social problems, and selecting appropriate behavioral responses.
- Sessions 17–24 target empathy and cognitive planning, through activities that require perspective-taking, identification of others’ emotions using contextual cues, anticipation of needs and actions, and selection of appropriate tools or steps to achieve a goal.

Although sessions were organized around specific skill domains, tasks were intentionally designed to engage multiple cognitive and socio-emotional processes simultaneously, reflecting the integrated nature of self-regulation and social functioning in early childhood.

#### Pedagogical and game design features

All activities were designed to be age-appropriate, visually engaging, and play-based, using familiar settings (e.g., home, classroom, park, zoo, beach) and everyday scenarios relevant to preschool children. Tasks incorporated elements such as drag-and-drop actions, touch-based selection, sequencing, and simple decision-making. Difficulty levels increased gradually within and across sessions by manipulating stimulus complexity, number of elements, and cognitive load.

The game also included explicit emotion regulation strategies, such as guided breathing exercises and calming routines, which were practiced repeatedly across sessions to promote skill consolidation and transfer to real-life situations.

#### Implementation support and monitoring

Sessions were implemented in the classroom with the presence of research assistants, who provided general instructions at the beginning of each session and technical support when needed, without offering task-specific guidance. At the end of each session, the research assistants collected the tablets and headphones and instructed the children to pay attention to the final remarks and questions about what was played during the session, what they learned, and how they could apply the learning in their daily activities. Early years educators were present during sessions to support classroom management and student motivation but did not directly intervene in task completion.

A web-based dashboard allowed educators, parents, and the research team to monitor session completion, activity progression, and basic performance indicators, supporting intervention monitoring and adherence to the intervention schedule.

A detailed description of each session and activity is provided in Supplementary File 1 and Screenshots in Supplementary File 2.

### Control

Schools allocated to the control group continued with their standard preschool curriculum (treatment as usual), as defined by the national educational guidelines for early childhood education issued by the Chilean Ministry of Education [66]. No additional intervention, training, or materials related to Japi 2.0 were provided to control schools during the study period.

The standard curriculum includes pedagogical activities aimed at promoting children’s overall development, including aspects related to emotion regulation, social interaction, autonomy, and early cognitive skills, which are typically delivered through routine classroom practices led by early years educators. These activities are not delivered through a manualized, structured, or technology-based program and may vary across schools and classrooms depending on institutional resources and teaching practices.

Control schools did not receive tablet-based activities related to the Japi 2.0 platform and did not have access to the game or the associated web-based dashboard during the trial. Schools in the control group were offered the possibility of receiving information about the intervention after completion of the study.

This control condition was selected to reflect real-world educational practice, allowing the feasibility and acceptability of implementing Japi 2.0 to be evaluated in comparison with usual preschool education rather than against an alternative structured intervention. However, given the small number of control clusters, variability in standard preschool practices across schools and classrooms may represent an important source of outcome variability.

## Measures

### Primary outcomes

#### Acceptability of the Japi 2.0 video game perceived by Educators

The primary outcome of this study was the acceptability of the Japi 2.0 video game, as perceived by early years educators involved in the implementation of the intervention. Acceptability was defined as the extent to which the video game was perceived as appropriate, engaging, and suitable for use with preschool children in the classroom context.

Acceptability was assessed at the end of the intervention period using a brief, study-specific questionnaire completed by early years educators. The questionnaire included seven items rated on a 4-point Likert scale, ranging from 1 = Totally disagree to 4 = Totally agree, covering the following dimensions:

1. Overall attractiveness of the game’s design.
2. Engagement of the activities presented in each session.
3. Appropriateness of the game contents for the age of the students.
4. Suitability of the activity dynamics for the students.
5. Perceived capacity of the game contents to stimulate cognitive skills.
6. Perceived capacity of the game contents to stimulate socio-emotional skills.
7. Overall level of children’s involvement and engagement with the game.

These items were selected to capture educators’ perceptions of the video game’s design, pedagogical relevance, developmental appropriateness, and observed student engagement during implementation. Descriptive statistics, including response frequencies, means and standard deviations, medians, and interquartile ranges, were used to summarize educators’ responses. Results are presented in Table 2.

#### Acceptability of the Japi 2.0 video game perceived by students

Acceptability of the Japi 2.0 video game from the children’s perspective was assessed at the end of the intervention using a brief, assisted questionnaire administered individually to participating students. Given the young age of the children, items were presented orally by a trained research assistant, using simple language and visual response options to facilitate comprehension.

The student acceptability questionnaire consisted of five items, each rated on a 5-point Likert-type scale, capturing children’s subjective experience with the video game and its delivery format. The items assessed the following aspects:

1. Enjoyment of the Japi video game (“Did you like the Japi video game?”).
2. Emotional experience while playing the game (“How did you feel when you played the Japi game?”).
3. Emotional experience when playing with a tablet (“How did you feel playing with a tablet?”).
4. Emotional experience when wearing headphones during gameplay (“How did you feel wearing the hearing aids?”).
5. Self-perceived change in behavior after participating in the Japi intervention, compared to before playing the game (“Comparing your behavior before you first played the Japi game with your behavior now, you would say that you are…”).

For the first four items, response options ranged from 1 = Very sad to 5 = Very happy. For the item assessing perceived behavioral change, response options ranged from 1 = Much worse to 5 = Much better.

Descriptive statistics, including means, standard deviations, medians, and interquartile ranges, were used to summarize students’ responses. Results are presented in Table 3.

#### Acceptability of the Japi web-based dashboard

Acceptability of the Japi web-based dashboard, designed to allow parents and early years educators to review students’ progress and information about the skills stimulated by the video game, was assessed at the end of the intervention period using study-specific questionnaires.

##### Acceptability perceived by parents

Parents’ acceptability of the Japi website was assessed using a self-administered questionnaire composed of seven items rated on a 4-point Likert scale (1 = Totally disagree to 4 = Totally agree). The items evaluated parents’ perceptions regarding usability, clarity, usefulness, and applicability of the information provided on the platform. Specifically, the questionnaire assessed whether parents:

1. Were able to navigate the website smoothly and without difficulty.
2. Were able to allocate time to review the website without compromising other tasks.
3. Could easily understand the information presented.
4. Found the information useful for understanding the definitions of each skill stimulated by Japi.
5. Found the information helpful for learning about their child’s progress in the game.
6. Found the information helpful for reinforcing skills stimulated through play in their child.
7. Found the information helpful for reinforcing parenting strategies at home.

Descriptive statistics, including response frequencies, means, standard deviations, medians, and interquartile ranges, were used to summarize parents’ responses. Results are presented in Table 4.

##### Acceptability perceived by early years educators

Early years educators’ acceptability of the Japi website was assessed using a parallel study-specific questionnaire, also rated on a 4-point Likert scale (1 = Totally disagree to 4 = Totally agree). The questionnaire included seven items assessing educators’ perceptions of the dashboard’s usability and pedagogical usefulness. Educators were asked whether they:

1. Were able to navigate the website smoothly and without difficulty.
2. Were able to review the website information within their working hours.
3. Found the information helpful for planning classroom activities.
4. Could easily understand the information presented.
5. Found the information useful for understanding the definitions of each skill stimulated by Japi.
6. Found the information helpful for learning about their students’ progress in the game.
7. Found the information useful for reinforcing specific skills in students.

Descriptive statistics were used to summarize educators’ responses, and results are reported in Table 4.

These questionnaires were specifically developed for the purposes of this pilot feasibility study and were intended to obtain descriptive information regarding perceived usability, engagement, and satisfaction. They were not designed as standardized psychometric instruments, and therefore findings should be interpreted as subjective perceptions rather than as validated measures of acceptability.

#### Feasibility

Feasibility was defined as the extent to which the Japi 2.0 intervention could be implemented as planned within routine preschool settings. Feasibility indicators were pre-specified to capture key implementation and study process parameters relevant to the design of a future definitive trial.

Feasibility was assessed across four main domains:

##### Recruitment and retention

Feasibility of recruitment and retention was evaluated at both the school and student levels. Indicators included the number of schools invited and enrolled, the number of schools randomized, the number of eligible students approached, consented, and retained at baseline and post-intervention follow-up. These indicators were derived from administrative records and summarized using a CONSORT-style flow diagram.

##### Intervention delivery and implementation

Feasibility of intervention delivery was assessed by documenting whether the planned number of sessions was delivered within the intended time frame. Indicators included the number of sessions scheduled, the occurrence of session rescheduling, and reasons for rescheduling, as recorded in standardized implementation logs maintained by the research team.

##### Student attendance and dose received

Student-level feasibility was assessed through attendance records, used as a proxy for dose received. Attendance was operationalized as the number of Japi sessions attended by each student across the intervention period and categorized into pre-defined ranges corresponding to low, moderate, and high exposure.

##### Data completeness

Feasibility of data collection was evaluated by examining the completeness of primary and secondary outcome measures at baseline and post-intervention assessments. Indicators included the proportion of participants with available data for each outcome and time point, based on study records.

All feasibility indicators were summarized descriptively to inform the refinement of recruitment procedures, intervention delivery, outcome selection, and sample size estimation for a future fully powered randomized controlled trial.

Although formal a priori feasibility thresholds were not predefined, feasibility indicators were interpreted in relation to commonly used benchmarks in pilot and feasibility studies [61]. In particular, high levels of session attendance (e.g., participation in the majority of scheduled sessions), full completion of planned intervention sessions, and adequate data completeness at follow-up were considered indicative of acceptable feasibility and supportive of progression to a future definitive trial.

### Secondary outcomes

#### Working memory

We used two measures:

1. Corsi Blocks Test [67]. This test assesses visuospatial short-term working memory. It involves repeating a sequence of up to nine identical spatially separated blocks on a screen. The sequence starts out simple but becomes more complex until the subject’s performance begins to decline. It has shown good psychometric characteristics among school children with a reliability mean coefficient of 0.74 (odd-even corrected by the Spearman-Brown) [68]. Pre-kindergarteners take around 7 min to complete this test.
2. Auditive Working Memory test [69]. A sequence of audio messages of increased difficulty is presented, and the child is requested to remember these messages. The test is completed when the child has attempted to repeat the full set of 21 messages. It has shown good psychometric characteristics with reliability correlation coefficients for test-retest scores of 0.77 for 5-year-old children [69]. Pre-kindergarteners take around 5 min to complete this test.

#### Inhibitory control

We used the Hearts and Flowers task [70], a hybrid combining elements of Simon and spatial Stroop tasks. This test consists of three consecutive tasks; in the first block, the person must press a key on the same side on which a heart appears (congruent block); in the second task, he/she must press a key on the opposite side of which a flower appears (incongruent block). Finally, there is a mixed block in which hearts and flowers appear randomly. The individual must follow two rules simultaneously, depending on the stimulus that appears, forming a mixed block [56]. It requires working memory and inhibition control. Pre-kindergarteners take around 10 min to complete this test.

#### Cognitive planning

We used The Tower of London developed by Shallice (1982), based on the pre-existing Tower of Hanoi [54]. This instrument is applied to children through individual interviews. The participant’s task is to convert the initial position of the balls to the position demonstrated by the evaluator. Ball movements are limited by the fact that pegs differ in terms of the maximum number of balls they can hold at any time, and participants cannot move more than one ball at a time. There are 12 tasks. We used the score obtained by summing the number of times children answered each task correctly (score from 0 to 12). The higher the score, the better the skill. It has shown good psychometric characteristics among school children, with a correlation to other executive function tools with Pearson coefficients ranging from 0.40 to 0.48 [54]. Pre-kindergarteners take around 12 min to complete this test.

#### Emotion recognition

We used the Assessment of Children’s Emotions Skills (ACES) [71]. It consists of facial expressions task aiming to evaluate emotion expression knowledge and whether the subjects exhibit any anger bias. The 26-item scale consists of colour photographs of ethnically diverse elementary schoolchildren depicting four expressions of each of the four basic emotions (happy, sad, angry, and scared) and 10 images of children without obvious facial expressions [72, 73]. The examiner shows the child the photographs one at a time and each time asks, “Is the child in the picture happy, sad, angry, or scared?” Then, the examiner registers the child’s answer. The emotion accuracy score reflects how many items the children answer correctly, and the anger bias score is the percentage of time the children incorrectly identify the faces as displaying anger [49]. Recently, it has shown acceptable psychometric characteristics with reliability among 6-year-old children with Cronbach’s α 0.64 [74]. Pre-kindergarteners take around 10 min to complete this test.

#### Social competence

We used the Challenging Situations Task (CST) [75]. This instrument evaluates the ability of children to solve social problems. The children are presented six vignettes that describe problems between peers. Following the presentation of each challenging situation, four pictures of happy, sad, angry, and neutral affect are presented in random order. The child is asked to point to the picture that best describes how they feel when [this situation] happens. Then, four pictures of behavioral responses (prosocial, aggressive, manipulation of others’ feelings, and avoidant) are presented in random order, and the child is asked, “What do you do [in this situation]?” The answers are categorized into four possibilities: prosocial, aggressive, cry, and avoidant. Scores for affective and behavioral responses used are the number of times each affect, and each behavioral response is chosen by each child across the six situations. It has shown acceptable psychometric characteristics of internal consistency with mean inter-item correlations among 4-year-old children ≥ 0.29 [75]. We used the prosocial subscale in this report, which indicates the number of times children chose the prosocial option in each situation (score from 0 to 6). Pre-kindergartners take around 10 min to complete this test.

#### Empathy

We used The Empathy Scale for Children (ESC) [53], a tool developed for measuring children’s empathic skills. The scale consists of 12 essays with picture cards about four basic emotions (happiness, sadness, anger and fear) and cards about facial expressions, where, for example, a facial expression of a character is missing under a situation, and the evaluated child matches this with emotion. The higher the score, the better the skill. It has shown good psychometric characteristics with reliability correlation coefficients for test-retest scores of 0.89 for 4 to 6-year-old children [53]. Pre-kindergarteners take around 5 min to complete this test.

#### Behavioral problems and psychological assessment

We used four measures:

1. The Strengths and Difficulties Questionnaire (SDQ) [76] is widely used in Chile [77, 78].This 25-question questionnaire explored different symptoms grouped into five sub-scales (with five items each): [1] emotional symptoms [2], behavioral problems [3], problems with peers [4], symptoms of lack of attention and hyperactivity, and [5] prosocial skills. The first four sub-scales referred to difficulties that children may have and could be grouped together in a general difficulties sub-scale (20 items). The prosocial skills sub-scale referred to positive and adaptive behaviors in relationships with others. Each item was answered on a response scale ranging from 1 = not true to 3 = absolutely true. There were versions for educators and parents (to evaluate children aged 4 to 16 years) and a self-report version for adolescents (aged 11 to 16 years). The SDQ had been widely used [79] and had shown good psychometric characteristics, with a Cronbach’s α of 0.73 [80]. The educators’ and parents’ versions of this instrument were used.
2. The Eyberg Child Behavior Inventory (ECBI) [81] is a behaviorally specific rating scale that assesses the frequency and severity of disruptive behaviors in the home setting and the extent to which parents find these behaviors troublesome. It provided two measures: the frequency of disruptive behaviors (Intensity Score), rated on 7-point scales, and the number of disruptive behaviors identified by parents as problematic (Problem Score). The ECBI demonstrated high internal consistency for both the Intensity Scale (*r* = 0.95) and the Problem Scale (*r* = 0.94) [81, 82]. By evaluating the variety and frequency of behaviors commonly exhibited by children, the instrument distinguished normal behavior problems from conduct-disordered behavior in children and adolescents. The established cutoff scores of 127 for the Intensity Scale and 11 for the Problem Scale were valid [83].
3. The Social Competence Scale – Parent Version is a 12-item instrument designed to evaluate children’s prosocial behaviors, communication skills, and self-regulation [84]. Each item described a behavior that could be observed in social contexts, such as the child’s ability to express opinions without being domineering or to regain calm when overly excited. Parents rated the extent to which each statement reflected their child’s behavior using a five-point Likert scale ranging from 0 (not at all) to 4 (very well). The scale comprised two subscales: Prosocial and Communication Skills (Items 4, 7, 9, 10, 11, and 12) and Emotional Regulation Skills (Items 1, 2, 3, 5, 6, and 8). In addition to these subscale scores, an overall score based on all 12 items was calculated. Subscale and total scores were derived from the mean of item responses. Scores were not calculated when more than half of the items within a subscale or the total scale were missing, and measures with entirely missing responses were excluded from scoring. Internal consistency analyses (Cronbach’s alpha) indicated that both the total score and the subscale scores were reliable [84].
4. Perceived Behavioral and Self-Regulation Change Questionnaire (PBSRC-Q): Parents and early years educators completed a study-specific questionnaire developed by the research team to assess their perception of change in children’s executive functions, self-regulation skills, and problem-solving abilities following participation in the intervention. This questionnaire was designed to complement standardized behavioral screening by capturing context-specific and functional changes that may not be fully reflected in symptom-based measures. The questionnaire included seven items, rated on a 4-point Likert scale (1 = Totally disagree to 4 = Totally agree), assessing perceived changes in: (1) emotion regulation; (2) empathy toward others’ emotions; (3) problem-solving with peers; (4) planning abilities to achieve goals; (5) concentration on school activities; (6) behavioral control (e.g., impulsivity); and (7) overall behavior in the home or classroom context, depending on the respondent. Parallel versions of the questionnaire were administered to parents and early years educators, with wording adapted to reflect observations in the home and school settings, respectively. Responses were summarized using descriptive statistics, and results are presented in Table 8.

### Data analysis

General school features (number of Pre-kindergarten classes and number of students per class) were used to randomize. Additionally, descriptive statistics (sex, age in months, and secondary outcomes scores) were used to compare the two arms at baseline.

#### Primary outcomes

Acceptability. We described the frequencies of the degree of agreement with several statements by Early Year Educators regarding acceptability in the following aspects: general and implementation acceptability, usefulness, and satisfaction.

Feasibility. We present the number of schools and students participating in each study stage and the response rates for the baseline and after-intervention surveys. In addition, we present the frequencies of a number of sessions played and attendance.

#### Secondary outcomes

Analyses of secondary outcomes were conducted using available outcome data from randomized participants within a multilevel modelling framework. Adjusted Mean differences and p-values were calculated as measures of association between the experimental condition (Intervention arm) and the behavioral outcome. Secondary outcome analyses were conducted using multilevel linear regression models to account for the hierarchical structure of the data (students nested within schools). Models included a random intercept for school and fixed effects for study arm, baseline scores of the outcome variable, sex, and age. Adjusted mean differences and 95% confidence intervals were estimated to emphasize the magnitude and uncertainty of effects rather than confirmatory hypothesis testing. Given the small number of randomized clusters, analyses were considered exploratory and interpreted cautiously. Model convergence was assessed during estimation, and all specified models converged adequately. Given baseline imbalances, we have included sex and age in the adjusted models. Adjusted mean differences and 95% confidence intervals are presented to emphasize estimation and uncertainty rather than confirmatory hypothesis testing. Because this pilot study involved a small number of clusters and limited missing data across most outcomes, no multiple imputation procedures were implemented. Therefore, analyses were based on available outcome data for each measure within the multilevel modelling framework.

Given the small number of clusters included in this pilot study and the number of domains assessed, analyses of secondary outcomes were considered exploratory and were not intended to provide confirmatory evidence of effectiveness. Accordingly, emphasis was placed on estimating the direction and magnitude of change rather than on formal hypothesis testing. Findings should therefore be interpreted as preliminary signals that may inform the selection of primary outcomes and effect size estimates for a future fully powered trial [64, 65].

Data was analysed with Stata 17.0.

## Results

### Sociodemographic features

Table 1 presents baseline demographic characteristics and secondary outcome measures for the control group (CG; *n* = 62), the JAPI 2.0 intervention group (*n* = 100), and the total sample. Mean age was comparable across groups, with an overall mean of 55.7 months. The proportion of females was higher in the JAPI 2.0 group compared with the CG (71.0% vs. 53.2%), and females represented 64.2% of the total sample.

Baseline secondary outcome measures were generally similar between groups. Emotional skills assessed with ACES, social competence measured by the Challenging Situations Task (CST), and empathy scores showed comparable mean values in the CG and JAPI 2.0 groups. Executive function outcomes—including inhibitory control (Hearts & Flowers), visual working memory (Corsi’s Cubes), auditory working memory (Woodstock), and planning (Tower of London)—also demonstrated comparable baseline levels across groups.

Regarding behavioral outcomes, teacher- and parent-reported Strengths and Difficulties Questionnaire (SDQ) total difficulties scores were similar between groups at baseline. The parent-reported Eyberg Child Behavior Inventory (ECBI) Intensity and Problem scores were also within comparable ranges across groups. Finally, baseline scores on the Social Competence Scales were reported for both groups, with slightly higher mean values in the JAPI 2.0 group.

Overall, Table 1 indicates that the control and intervention groups were broadly comparable at baseline across demographic variables and secondary outcome measures.

**Table 1:** Students’ baseline features

Variable		CG (*n*=62)		JAPI 2.0 (*n*=100)		Total
	*n*	% / Mean (SD)	*n*	% / Mean (SD)	*n*	% / Mean (SD)
Age (months)	59	54.9 (4.04)	96	55.2 (3.3)	155	55.1 (3.6)
Sex						
Female	33	53.2	71	71	104	64.2
Male	29	46.8	29	29	58	35.8
Secondary outcomes						
ACES	57	11.96 (2.37)	96	12.08(2.95)	153	12.03(2.73)
CST (Social competence)	57	2.84 (1.40)	96	2.49 (1.64)	153	2.6(1.6)
Empathy (CES)	56	6.23(2.27)	96	6.38(2.14)	152	6.32(2.2)
Inhibitory control (H&F)	57	24.0(8.17)	96	24.95(7.10)	153	24.6(7.5)
Working memory (Visual)(Corsi´s Cubes)	58	1.86(1.15)	96	1.93(1.25)	154	1.9(1.2)
Working memory (Audio)(Woodstock)	57	1.32(1.40)	96	1.66(1.83)	153	1.52(1.7)
Planning (Tower of London)	54	4.63(3.84)	92	5.57(3.25)	144	4.9 (3.6)
SDQ Teachers	65	9.43(3.08)	88	7.98(2.29)	156	8.6(2.7)
SDQ Parents	51	9.82(2.47)	65	10.03(2.76)	116	9.9(2.6)
ECBI Intensity - Parents	60	88.98(30.03)	65	92.76(39.66)	125	90.95(35.28)
ECBI Problems - Parents	41	7.34(8.83)	55	7.56(8.07)	96	7.46(8.36)
Social Competence Scales	59	25.38(10.10)	64	29.45(9.02)	123	27.50(9.73)

*CG *Control Group, *Intervention Group* JAPI 2.0, *SD *Standard Deviation, *ACES *Assessment of Children’s Emotional Skills, *CST* Challenging Situations Task, *CES* The Empathy Scale for Children, *H&F* Hearts and Flowers Task, *SDQ* Strengths and Difficulties Questionnaire, *ECBI* The Eyberg Child Behavior Inventory

### Primary outcomes

#### Acceptability of Japi 2.0 by educators and students

Educators reported high acceptability of the Japi 2.0 intervention across all assessed dimensions. All items showed median ratings of 3 on a 1–4 scale, indicating agreement, with interquartile ranges between 3 and 4. Educators consistently perceived the game’s design, activities, and content as attractive and developmentally appropriate, and noted that the sessions supported both cognitive and socio-emotional skill development. They also observed that children were generally engaged throughout the intervention. See Table 2.

**Table 2:** Acceptability perceived by educators

	Response scores											
	1		2		3		4		Total			
Acceptability	n	%	n	%	n	%	n	%	n	Mean (SD)	Median	IQR
Overall, the game’s design is attractive (1-4)^a^	0	0	0	0	2	50	2	50	4	3.5 (0.57)	3.5	(3-4)
The activities presented in each session were engaging (1-4)	0	0	0	0	3	75	1	25	4	3.25 (0.5)	3	(3-4)
The contents of the game are appropriate for the age of my students (1-4)	0	0	0	0	2	50	2	50	4	3.5 (0.57)	3.5	(3-4)
The dynamics of the activities were suitable for my students (1-4)	0	0	0	0	3	75	1	25	4	3.25 (0.5)	3	(3-3.25)
The contents of the game allow to stimulate cognitive skills (1-4)	0	0	0	0	1	25	3	75	4	3.75 (0.5)	4	(3.75-4)
The contents of the game allow to stimulate socio-emotional skills (1-4)	0	0	0	0	1	25	3	75	4	3.75 (0.5)	4	(4-4)
In general, the children were involved and engaged with the game (1-4)	0	0	1	25	2	50	1	25	4	3 (0.81)	3	(2.5-3.5)

Four out of six early educators responded to the acceptability survey. *SD* Standard Deviation, *IQR* Interquartile Range

^a^Response answers from 1 to 4 mean: 1= Totally Disagree, 2= Disagree, 3= Agree, and 4= Totally Agree

Table 3 presents students’ ratings of the acceptability of the JAPI video game. Overall, mean scores indicate high levels of enjoyment with the game and positive experiences using the tablet, as well as favorable emotional responses while playing. Students also reported perceived improvements in their behavior after engaging with the game.

**Table 3:** Acceptability perceived by students

	Total				
	*n*	Mean	SD	Median	IQR
Did you like the video game JAPI? (1-5)^a^	92	4.6	0.71	5	1
How did you feel when you played the JAPI game? (1-5)	92	4.2	1.03	5	1
How did you feel playing with a tablet? (1-5)	92	4.4	0.84	5	1
How did you feel wearing the hearing aids? (1-5)	92	3.9	1.23	5	2
Comparing your behavior before you first played the game Japi with the behavior you have now, you would say that you are… (Much worse – much better) (1-5)^b^	92	4.4	1.04	5	1

*SD* Standard Deviation, *IQR* Interquartile Range

^a^Answers alternatives are 1=Very sad; 2=Sad; 3=Indifferent; 4=Happy; 5=Very happy

^b^Answers alternatives are 1=Much worse; 2=Worse; 3=Indifferent; 4=Better; 5= Much better

#### Acceptability of web-based dashboard by educators and parents

Table 4 summarizes parents’ and educators’ perceptions of the JAPI website. Among parents (*n* = 52), responses indicated high levels of acceptability and perceived usefulness. Most parents agreed or totally agreed that the website was easy to navigate, that the information was clear and understandable, and that it could be reviewed without interfering with other daily tasks. Parents also reported that the information was useful for understanding the skills stimulated by JAPI, monitoring their child’s progress in the game, reinforcing play-stimulated skills, and supporting parenting strategies at home, with median scores of 3 across all items.

Educators’ perceptions (*n* = 4 respondents) also reflected favorable acceptability. Most educators agreed or totally agreed that the website was easy to navigate, understandable, and useful for learning about the skills stimulated by JAPI. The information was perceived as helpful for monitoring students’ progress and for reinforcing specific skills in the classroom. Median scores ranged from 3 to 3.5 across items, indicating overall positive perceptions despite the small number of respondents.

**Table 4:** Parents’ and Educators’ perception JAPI website

	Response scores												
	1		2		3		4		Total				
Perception perceived by Parents	n	%	n	%	n	%	n	%	n	Mean	SD	median	IQR
I was able to navigate the website smoothly and without difficulty (1-4)^a^	1	1.92	8	15.38	30	57.69	13	25.00	52	3.05	0.69	3	(0.5)
I was able to allocate time to review the website without compromising or reducing the time spent on other tasks or tasks (1-4)	1	1.92	7	13.46	35	67.31	9	17.31	52	3	0.62	3	(0)
I was able to easily understand the information presented (1-4)			9	17.31	31	59.62	12	23.08	52	3.05	0.63	3	(0)
The information was useful to know the definitions about each skill stimulated by Japi (1-4)	1	1.92	6	11.54	33	63.46	12	23.08	52	3.07	0.65	3	(0)
The information was helpful in learning about my child’s progress in the game (1-4)	2	3.85	5	9.62	30	57.69	15	28.85	52	3.11	0.7	3	(1)
The information was helpful in reinforcing play-stimulated skills in my child (1-4)	1	1.92	5	9.62	31	59.62	15	28.85	52	3.15	0.66	3	(1)
The information was helpful in reinforcing parenting strategies at home (1-4)	1	1.92	7	13.46	32	61.54	12	23.08	52	3.05	0.66	3	(0)
Helpfulness perceived by educators^**b**^													
I was able to navigate the website smoothly and without difficulty (1-4)	0	0	1	25	1	25	2	50	4	3.25	0.95	3.5	(1.5)
I was able to review the website information within my business hours (1-4)	0	0	1	25	1	25	2	50	4	3.25	0.95	3.5	(1.5)
The information was helpful in planning classroom activities (1-4)	0	0	1	25	3	75	0	0	4	2.75	0.5	3	(0.5)
I was able to easily understand the information presented (1-4)	0	0	1	25	2	50	1	25	4	3	0.82	3	(1)
The information was useful to know the definitions of each skill stimulated by Japi (1-4)	0	0	0	0	3	75	1	25	4	3.25	0.5	3	(0.5)
The information was helpful in learning about my students’ progress in the game (1-4)	0	0	1	25	3	75	0	0	4	2.75	0.5	3	(0.5)
The information was useful to be able to reinforce certain skills in students (1-4)	0	0	0	0	4	100	0	0	4	3	0	3	(0)

*SD* Standard Deviation, *IQR* Interquartile Range

^a^Response answers from 1 to 4 mean: 1= Totally Disagree, 2= Disagree, 3= Agree, and 4= Totally Agree

^b^Four out of six early educators responded to the acceptability survey

#### Session attendance by students

Table 5 presents students’ attendance across the JAPI video game sessions within the intervention group (*n* = 100). Most participants attended a high number of sessions, with 62% completing between 17 and 24 sessions. An additional 30% attended between 9 and 16 sessions, indicating moderate exposure to the intervention. Only a small proportion of students attended few or no sessions, with 6% participating in 1 to 8 sessions and 2% not attending any sessions.

**Table 5:** Students’ attendance during the Japi video game sessions

Range	*n*	%
0 sessions	2	2
1 to 8 sessions	6	6
9 to 16 sessions	30	30
17 to 24 sessions	62	62
Total	100	100

#### Implementation of sessions

Across the four intervention schools, a total of 144 scheduled sessions were planned, of which 34 (23.6%) required rescheduling; however, all 144 sessions (100%) were ultimately completed. Rescheduling ranged from 18.8% to 29.2% across schools. The main reasons for rescheduling were school-related events (41.2%), half-term holidays (26.5%), bank holidays (23.5%), and weather-related disruptions (8.8%). See Tables 6 and 7.

**Table 6:** Implementation of the Japi video game sessions

School		Scheduled sessions	Rescheduled sessions	Completed sessions
	*N*° of classes	*n* ^a^	*n* (%)	*n* (%)
1	1	24	7 (29.2%)	24 (100%)
2	1	24	5 (20.8%)	24 (100%)
3	2	48	13 (27.0%)	48 (100%)
4	2	48	9 (18.8%)	48 (100%)
Total	6	144	34 (23.6%)	144 (100%)

^a^The total number of sessions of the program was 24

Schools 1 and 2 implemented the program in class. Schools 3 and 4 implemented the program in 2 classes, so the total number of potential sessions was 48

**Table 7:** Reasons for session rescheduling

Reasons for rescheduling	*n* (%)
Half term holidays	9 (26.5%)
Bank holidays	8 (23.5%)
Weather conditions (heavy rains and flooding)	3 (8.8%)
Other reasons^a^	14 (41.2%)
Total	34 (100%)

^a^Among other reasons are: school celebrations (e.g., Teacher´s Day, School´s anniversary, National Day), assessments (e.g., local standard evaluations), off-campus teaching experiences (e.g., Visit to museum), and disturbances (e.g., community violent demonstrations)

### Helpfulness perceived by parents and educators

Parents reported improvements across multiple domains, with the majority agreeing or strongly agreeing that their children showed better emotional regulation, greater empathy, enhanced problem-solving with peers, and improved planning, focus, behavioral control, and overall behavior at home.

Educators also indicated positive changes, noting that children demonstrated better emotional regulation, empathy, problem-solving, planning, concentration, behavioral control, and general classroom behavior, with most ratings falling in the higher agreement categories. See Table 8.

**Table 8:** Parents’ and Educators’ perception of improvement in executive functions, self-regulation skills, and problem-solving

	Response score												
	1		2		3		4		Total				
Helpfulness perceived by Parents	n	%	n	%	n	%	n	%	n	Mean	SD	median	IQR
My child can more easily regulate their emotions (1-4)^a^	2	3.85	11	21.15	30	57.69	9	17.31	52	2.9	0.7	3	(2.5-3)
My child increased their empathy with the emotions of others (1-4)	3	5.77	3	5.77	29	55.77	17	32.69	52	3.2	0.8	3	(3-4)
My child improved in problem solving with peers (1-4)	2	3.85	6	11.54	37	71.15	7	13.46	52	2.9	0.6	3	(3)
My child has improved in their ability to plan their actions to achieve a goal (1-4)	1	1.92	8	15.38	31	59.62	12	23.08	52	3.0	0.7	3	(3)
My child has improved in his or her ability to focus on school activities (1-4)	4	7.69	2	3.85	30	57.69	16	30.77	52	3.1	0.8	3	(3-4)
My child has improved control of his/her behavior (e.g., decreased impulsivity) (1-4)	5	9.62	5	9.62	30	57.69	12	23.08	52	2.9	0.8	3	(3)
Overall, your child has improved behavior at home (1-4)	5	9.62	6	11.54	24	46.15	17	32.69	52	3.0	0.9	3	(3-4)
Helpfulness perceived by educators^b^													
Children can regulate their emotions more easily (1-4)	0	0	1	20	3	60	1	20	5	3	0.7	3	(3-4)
Children increased their empathy with the emotions of others (1-4)	0	0	1	20	2	40	2	40	5	3.2	0.8	3	(3-3.5)
Children improved in problem-solving with their peers (1-4)	0	0	1	20	2	40	2	40	5	3.2	0.8	3	(3-4)
Children have improved in their ability to plan their actions to achieve a goal (1-4)	0	0	1	20	3	60	1	20	5	3	0.7	3	(3-3.5)
Children have improved in their ability to concentrate on school activities (1-4)	0	0	2	40	3	60	0	0	5	2.6	0.5	3	(2-3)
Children have improved control of their behavior (e.g., less impulsivity) (1-4)	0	0	1	20	3	60	1	20	5	3	0.7	3	(3-3)
In general, children have improved their behavior in the classroom (1-4)	0	0	1	20	3	60	1	20	5	3	0.7	3	(2.5-3.5)

*SD* Standard Deviation, *IQR* Interquartile Range

^a^Response answers from 1 to 4 mean: 1= Totally Disagree, 2= Disagree, 3= Agree, and 4= Totally Agree

^b^Five out of six early educators responded to the acceptability survey

No adverse events, intervention-related distress, or unintended negative consequences associated with participation in Japi 2.0 were reported by schools, educators, parents, or children during the study period. Minor technical issues (e.g., occasional audio or tablet connectivity problems) were infrequent and resolved during implementation without interrupting session completion.

### Exploratory secondary outcomes

Table 9 presents exploratory secondary outcome measures at baseline and post-intervention in the control and JAPI 2.0 intervention groups, together with adjusted between-group estimates. Analyses were adjusted for baseline scores and clustering.

For emotional and social outcomes, both groups showed increases from baseline to post-intervention in emotion recognition (ACES) and social competence assessed with the CST; however, adjusted between-group differences were not statistically significant. In contrast, empathy scores increased in both groups, with a statistically significant adjusted between-group difference favoring the intervention group (adjusted mean difference = 0.84, 95% CI 0.06 to 1.62, *p* = 0.038).

Regarding executive functions, improvements from baseline to post-intervention were observed in inhibitory control, visual and auditory working memory, and planning in both groups. Nevertheless, none of the adjusted between-group comparisons for these outcomes reached statistical significance.

For behavioral outcomes, teacher-reported SDQ total difficulties scores decreased modestly from baseline to post-intervention in both groups, with no statistically significant adjusted between-group differences. In contrast, parent-reported SDQ total difficulties showed a greater reduction in the intervention group compared to the control group in adjusted analyses (adjusted mean difference = − 0.84, 95% CI − 1.41 to − 0.26, *p* = 0.012). Parent-reported ECBI Intensity and Problem scores also decreased over time in both groups, although adjusted between-group differences were not statistically significant.

Finally, parent-reported social competence scores increased from baseline to post-intervention in both groups, with no statistically significant adjusted between-group difference.

**Table 9:** Secondary outcomes

	Control						Intervention							
	Baseline			Post-intervention			Baseline			Post-intervention			Adjusted^a^	
Measures	n	Mean	SD	n	Mean	SD	n	Mean	SD	n	Mean	SD	Mean difference (95% CI)	p-value
Emotion recognition (ACES)	57	11.96	2.37	55	12.89	2.69	96	12.08	2.95	94	13.26	1.48	+0.16 (-1.25 to 1.58)	0.785
Social Competence (CST)	57	2.84	1.40	55	3.18	1.71	96	2.49	1.64	94	3.16	1.64	+0.06 (-0.46 to 0.59)	0.998
Empathy (CES)	56	6.23	2.27	52	7.12	2.23	96	6.38	2.14	89	7.94	1.54	**+0.84 (0.06 to 1.62)**	**0.038**
Inhibitory control (H&F)	57	24.0	8.17	55	29.24	11.16	96	24.95	7.10	95	28.81	8.98	-1.59 (-7.10 to 3.91)	0.505
Working memory, visual (Corsi´s Cubes)	58	1.86	1.15	55	2.44	1.48	96	1.93	1.25	93	2.74	1.63	+0.27 (-0.17 to 0.71)	0.185
Working memory, audio(Woodstock)	57	1.32	1.40	55	1.95	2.53	96	1.66	1.83	93	3.35	3.82	+0.98 (-0.98 to 2.96)	0.267
Planning (Tower of London)	54	4.63	3.84	54	8.22	3.85	92	5.57	3.25	93	9.16	2.91	+0.10 (-2.76 to 2.97)	0.933
SDQ Total difficulties - Teachers	65	9.43	3.08	56	8.52	3.08	88	7.98	2.29	86	7.85	2.50	+0.15 (-0.86 to 1.16)	0.726
SDQ Total difficulties - Parents	51	9.82	2.47	42	9.60	2.60	65	10.03	2.76	39	8.85	2.12	**-0.84 (-1.41 to -0.26)**	**0.012**
ECBI Intensity - Parents	60	88.98	30.03	44	88.09	31.76	65	92.76	39.66	40	88.47	27.16	-8.25 (-20.48 to 3.99)	0.150
ECBI Problems - Parents	41	7.34	8.83	36	5.38	7.65	55	7.56	8.07	38	5.52	6.86	+0.33 (-4.47 to 5.13)	0.871
Social Competence - Parents	59	25.38	10.10	43	29.97	7.97	64	29.45	9.02	41	29.92	8.83	+0.69 (-2.05 to 3.44)	0.559

*SD* Standard Deviation,* ACES* Assessment of Children’s Emotions Skills,* CST* Challenging Situations Task,* CES* The Empathy Scale for Children,* H&F* Hearts and Flowers Task,* SDQ* The Strengths and Difficulties Questionnaire,* ECBI* The Eyberg Child Behavior Inventory

^a^ Analysis adjusted for baseline, sex, age (in months), and cluster score

## Discussion

The primary aim of this pilot randomized controlled trial was to evaluate the acceptability and feasibility of implementing Japi 2.0, a tablet-based gaming platform designed to stimulate cognitive and socio-emotional skills, in low-income preschool settings in Chile. The findings indicate that Japi 2.0 was highly acceptable to children, early years educators, and parents, and that the intervention could be delivered within routine preschool activities with adequate levels of participation and adherence. These results are particularly relevant given that poor acceptability and implementation barriers are among the most common reasons why early childhood interventions fail to achieve impact when scaled beyond controlled research environments [85, 86].

High levels of acceptability reported by educators are consistent with prior evidence showing that teachers’ perceptions of relevance, developmental appropriateness, and classroom fit are critical determinants of successful implementation of school-based interventions [87, 88]. Educators in this study consistently perceived Japi 2.0 as engaging, age-appropriate, and supportive of both cognitive and socio-emotional development, which aligns with studies indicating that play-based and game-based learning approaches are particularly well suited for early childhood education [23, 24]. The present findings suggest that, within a supported implementation model, Japi 2.0 could be integrated into routine preschool activities without major disruption to classroom dynamics [22].

Children’s own reports of enjoyment and positive emotional experiences while playing Japi 2.0 reinforce these findings. Enjoyment and intrinsic motivation may represent important conditions supporting sustained engagement with play-based interventions and may facilitate participation and implementation [59, 60]. However, the present study was not designed to directly evaluate the mechanisms through which engagement may influence developmental outcomes. Previous studies have shown that children are more likely to benefit from digital interventions when activities are perceived as playful and emotionally rewarding, rather than as explicit training or assessment tasks [25]. The favorable responses reported by children in the present study suggest that Japi 2.0 successfully achieved this balance.

Acceptability also extended to the web-based dashboard provided to parents and educators, who reported that the platform was easy to use, understandable, and helpful for monitoring children’s progress and reinforcing skills at home and in the classroom. This finding is consistent with evidence emphasizing the importance of caregiver involvement and transparency in early childhood interventions, particularly for promoting generalization of skills across contexts [89, 90]. Digital tools that facilitate communication between schools and families have been shown to enhance parental engagement and perceived usefulness of interventions, especially in socioeconomically disadvantaged settings [91]. Therefore, the web-based dashboard may represent a potentially useful implementation feature for supporting caregiver engagement and home–school communication. However, the present study did not directly evaluate whether dashboard use contributed to the generalization of skills or behavioral changes across contexts.

From a feasibility perspective, the study demonstrated that Japi 2.0 could be implemented largely as planned within real-world preschool settings. Most children attended a substantial proportion of sessions, indicating acceptable intervention “dose received,” which is a key feasibility indicator in early-phase trials [61]. Although session rescheduling was required due to school events and contextual constraints, all planned sessions were ultimately delivered. This pattern mirrors findings from other school-based interventions, where flexibility and adaptation to institutional rhythms are often necessary to maintain fidelity without compromising feasibility [92, 93]. An additional consideration is that the intervention was implemented with the support of research assistants, who supervised sessions and facilitated engagement. As such, the feasibility observed in this study reflects a supported delivery model rather than the standalone use of the digital platform. Future studies should examine the extent to which the intervention can be implemented with reduced external support, particularly in real-world educational settings.

Improvements observed across both intervention and control groups in several cognitive measures may reflect developmental maturation, practice effects associated with repeated testing, or increased familiarity with task demands over time, rather than intervention-specific effects [57, 58]. This possibility is particularly relevant in preschool-aged children, given the rapid developmental changes expected over relatively short periods [20, 21]. Accordingly, changes in cognitive performance should be interpreted cautiously within the context of this pilot study. Future trials would benefit from incorporating alternative test forms, longer retest intervals, and proximal indicators of in-game learning or performance trajectories to better distinguish intervention-related effects from retest and maturation processes.

Although most secondary outcomes did not show clear between-group differences, exploratory signals were observed for empathy and parent-reported emotional and behavioral difficulties. Specifically, children in the intervention group showed higher post-intervention empathy scores and lower parent-reported SDQ total difficulties compared to controls. However, these findings should be interpreted cautiously given the exploratory nature of the analyses, the number of outcomes assessed, the limited number of randomized clusters, and the reliance on unblinded parent reports. Accordingly, these results are best understood as preliminary and hypothesis-generating rather than as evidence of intervention effectiveness, and require confirmation in adequately powered trials [61, 62].

Several factors may further explain the pattern of findings. The limited sample size and cluster-randomized design constrained statistical power, particularly for detecting small effects after adjustment for baseline scores and clustering. Additionally, some higher-order executive functions, such as planning and working memory, may require longer exposure or more intensive practice to produce measurable changes, especially when assessed with standardized tasks rather than context-specific measures [94, 95]. It is possible that socio-emotional skills such as empathy may be more responsive to relatively brief, contextually embedded interventions; however, this interpretation remains highly preliminary given the exploratory nature of the analyses, the number of outcomes assessed, and the limited number of clusters.

### Limitations

This study has important limitations. As a pilot randomized controlled trial, it was not designed to establish efficacy, and all findings related to secondary outcomes should be considered exploratory. The small number of clusters and the limited number of educator respondents for some acceptability measures restrict generalizability. The small number of clusters, which constrains the stability of cluster-level variance estimates and limits the reliability of between-group comparisons. With few clusters per arm, randomization may not adequately balance confounders, and statistical inference at the cluster level becomes inherently unstable. Accordingly, findings related to secondary outcomes should be interpreted as exploratory and hypothesis-generating rather than as evidence of intervention effects.

Acceptability measures were based on study-specific questionnaires without formal psychometric validation. Although appropriate for an early-phase feasibility study, these measures primarily reflect subjective perceptions of usability and satisfaction rather than standardized constructs of acceptability. Furthermore, acceptability was assessed only after completion of the intervention, which limits the ability to distinguish sustained usability from potential novelty effects associated with exposure to a new digital platform. Future studies would benefit from repeated assessments of engagement and acceptability over time. There is a also limitation in the use of parent- and educator-reported outcomes, which were not blinded to group allocation. These measures may be influenced by expectancy or social desirability biases, particularly given the high levels of acceptability reported. As such, findings based on these informants should be interpreted as reflecting perceived changes rather than objective effects, and should be considered complementary to the assessor-blinded measures included in the study.

In relation to feasibility, because intervention sessions were delivered during regular school hours, session attendance was closely linked to overall school attendance. However, comparative attendance data across non-intervention school days or control classrooms were not systematically collected, limiting interpretation of whether absenteeism during intervention sessions differed from typical attendance patterns. Regarding attendance, variability in session attendance represented an additional limitation of the study. Most missed sessions were associated with routine school absenteeism or scheduling-related factors rather than intervention refusal; however, differences in exposure to the intervention may have influenced engagement and exploratory outcome findings. In addition, although intervention delivery was supervised by trained research assistants and all planned sessions were completed, formal quantitative measures of procedural fidelity were not collected. Future trials would benefit from incorporating standardized fidelity assessment procedures to more precisely evaluate implementation consistency across classrooms and schools. A further limitation is that formal a priori feasibility thresholds were not specified. Although feasibility indicators were interpreted using commonly accepted benchmarks, future studies would benefit from defining explicit progression criteria to support decision-making regarding scale-up and trial advancement.

Secondary outcomes were assessed immediately post-intervention, precluding conclusions about the sustainability of observed changes over time. In addition, the breadth of the assessment battery may have introduced participant burden, particularly given the young age of the sample. Although each measure has been previously used in child populations, the cumulative assessment time may have influenced performance due to fatigue or reduced attention, which should be considered when interpreting the findings. In addition, some psychometric evidence supporting the selected instruments derives from studies involving slightly older children, and therefore the validity and sensitivity of certain measures in preschool-aged populations should be interpreted cautiously.

An additional limitation relates to the use of a treatment-as-usual control condition across a small number of clusters. Variability in classroom practices and curricula between schools may have contributed to outcome differences independent of the intervention, thereby limiting the interpretability of between-group comparisons. Finally, the findings related to secondary outcomes should be interpreted with caution given the exploratory nature of the analyses, the number of outcomes assessed, and the limited number of randomized clusters. In this context, isolated statistically significant findings may reflect chance results rather than reliable intervention effects, particularly given the instability of statistical inference in very small cluster randomized trials. In addition, sample sizes varied across outcome measures due to incomplete data collection across assessments and informants, which may have further reduced precision and increased uncertainty. Because this was an early-phase pilot study with a small number of clusters, missing data procedures such as multiple imputation were not implemented, as these approaches may themselves yield unstable estimates in small samples. Accordingly, analyses should not be interpreted as a full intention-to-treat approach with imputed missing outcome data, but rather as exploratory available-case analyses conducted among randomized participants.

Despite these limitations, the study provides strong justification for further evaluation of Japi 2.0. The high levels of acceptability, session completion, attendance, and implementation feasibility observed in this pilot study support progression to a future adequately powered randomized controlled trial, in line with recommended frameworks for the development and evaluation of complex interventions [96, 97]. Future studies should include larger samples, longer follow-up periods, and refined outcome selection, as well as examine potential moderators and mediators of intervention effects. Strengthening integration with classroom practices and enhancing caregiver engagement through the digital dashboard may further increase the likelihood of meaningful and sustained impact. Additionally, these future trials may benefit from including an active tablet-based comparison condition to better control for nonspecific effects related to screen exposure, novelty, engagement, and adult attention during implementation.

## Conclusions

This pilot study demonstrates that Japi 2.0 is a feasible and acceptable digital intervention for preschool children in low-income educational settings. While evidence of efficacy remains preliminary, the findings are consistent with existing literature on early childhood, play-based, and digital interventions, and provide a solid empirical foundation for future large-scale evaluation aimed at promoting early cognitive and socio-emotional development and reducing mental health inequalities.

## Supplementary Information

Below is the link to the electronic supplementary material.

Supplementary Material 1.

Supplementary Material 2.

## Data Availability

The datasets generated and/or analyzed during the current study are not publicly available due to institutional data protection policies but are available from the corresponding author on reasonable request.
