# Supplementary material for: Japi 2.0, a gaming platform to stimulate cognitive and socio-emotional skills in early childhood: results of a pilot randomized controlled trial
Source: Child Adolesc Psychiatry Ment Health. 2026 Jul 21;20:145. doi: 10.1186/s13034-026-01133-1 (PMC13673852; doi:10.1186/s13034-026-01133-1)
Supplement: Supplementary file 1 [file 13034_2026_1133_MOESM1_ESM.docx]

**Suppl. 1: Description of each session of the Japi 2.0 video game**

#### Session 1

A ferret character appears on the screen to introduce the session by telling the children what they will be doing. This character will appear throughout the entire game.

**Activity 1:** Individually and continuously, fruits with faces showing different emotions will appear. The student must select the fruits that are happy from this set of fruits.

**Activity 2:** A cloud will appear with different situations representing the child's mind. The student must observe these situations and select the one that corresponds to the emotion requested by the ferret. The emotions are: happiness, sadness, anger, and fear.

**Activity 3:** In this activity, balloons will appear on the screen flying upwards (one at a time). The student must pop the balloons by touching them, according to the emotion requested. The emotions are: happiness, sadness, anger, and fear.

**Activity 4:** In this activity, different images of children expressing different emotions will appear. The student must select the face that corresponds to the emotion requested: happy, sad, or angry.

#### Session 2

As in session 1, the ferret character will appear on the screen to introduce the session by telling the children what they will be doing in the session.

**Activity 1:** Students must complete the face by dragging the eyes and mouth that represent the required emotion.

**Activity 2:** Individually and continuously, masks of various characters will appear on the screen. Students must only touch the masks of the characters that the audio asks them to according to the emotion they express. The emotions are: happy, sad, scared, or angry.

**Activity 3:** Students must play at painting on different faces, moving their finger over the face that appears on the screen. This will lead them to discover the emotion that the face conveys.

**Activity 4:** Several toothbrushes with faces showing different emotions will appear on the screen, individually and continuously. Students must touch the toothbrushes that have the requested emotion. The emotions are happy, sad, scared, or angry.

**Session 3**

**Activity 1:** The child must touch only the items that the friend in the game wants to buy at the kiosk. Several individual items will appear continuously (candy, patches, ice cream, drinks, among others.), and the child must touch only those of the color requested by the audio.

**Activity 2:** The ferret character will tell the children about different situations that happen to a child their age. Then, a set of balloons of different colors will appear on the screen. Students must pop the balloons (touch them) according to the color indicated by the audio in order to discover the different reasons for the situations.

**Activity 3:** Several items of different colors will appear on the screen. The child should touch the items only when they turn the color requested by the ferret (e.g., touch the toy boats when they turn orange).

**Activity 4:** The ferret character will tell the children about different situations that happen to a child their age. Then, a set of kites of different colors will appear on the screen. Students must touch the kites according to the color indicated by the audio in order to discover the different reasons behind the situations.

#### Session 4

For this session, the friends and the ferret arrive at a park to play.

**Activity 1:** A park appears with the following scene: a child holding another child by the arm, crying, who is halfway to falling. The latter has a skateboard tangled between his feet, and one of his shoes has untied laces. Very close to them, there is a protruding sidewalk, and next to it a bench.

The child must identify five possibilities related to why the child in the scene is sad. The student must touch the different options and the audio will narrate the reasons (e.g., he could have tripped over his untied shoe, he could have fallen off the skateboard, his friend could have pushed him and that's why he fell, among others.).

**Activity 2:** The face of a child appears on the screen, and next to it are several small objects that continuously change color. The game consists of touching only the objects of the color requested by the audio, depending on the friend's mood. If the friend is happy, they must touch the objects of one color; if they are sad, they must touch the objects of another color. For example: if the friend is happy, they must touch the red cars; if they are sad, they must touch the yellow cars.

**Activity 3:** In this activity, the child will be presented with five different scenes, where they must identify the negative emotion of one of the characters. Then they must choose a behavior (from 3 possible options) that could make the character with the negative emotion feel calm or happy. The situations are appropriate for the students' age: one classmate scares another, one child takes a toy from another, a child throws a tantrum at their mother in the supermarket, among others.

**Activity 4:** This exercise has two parts:

1. The objective of this activity is for all the faces presented to the student at the beginning to be happy at the end of the game. To do this, they must continuously touch the faces until they become happy.
2. Several elements will appear on the screen that constantly change color. The child must touch the elements only when they are the color that the game asks for (touch the pencils only when they are black). The difficulties of this activity lie in the fact that, on the one hand, there are several small elements on the screen and, on the other hand, these elements change color every so often.

#### Session 5

The session begins in a zoo setting.

**Activity 1:** For this activity, students must play at "catching" the insects requested by the audio. For each of the five trials, they must perform two actions:

1. From different options, select the most comfortable way to catch the insect requested (e.g., the most comfortable way to hold the net to catch butterflies. The ways to hold the net that are presented may be: with the mesh upside down, holding it by the handle, among others).
2. Select the insect of the color requested by the audio. To do this, a large insect will appear in the middle of the screen and two small insects below that will be used as buttons. The insect in the middle will change color, but the buttons will not. The child must press the button of the opposite color to the insect in the middle (e.g., if the large butterfly is red, press the blue butterfly button; if the large butterfly is blue, press the red butterfly button).

**Activity 2:** The child must look at a set of images showing different situations. Based on these, they must classify the images according to whether they are comfortable or uncomfortable situations. To do this, they will have to drag the comfortable situations to the blue column on the left of the screen and the uncomfortable ones to the pink column on the right of the screen.

**Activity 3:** In this activity, children must perform two actions:

1. Four faces are displayed on the screen, and they must select the face that has an enthusiastic expression (a version of happiness).
2. They must listen and memorize the combination of a key of a certain color and an animal. To do this, a large animal will appear in the middle of the screen and two keys of different colors will appear below, which will be used as buttons. The animal in the middle will change, but the keys will not. The child must press the button with the color that corresponds to each of the animals (e.g., lion, red button; giraffe, blue button).

**Activity 4:** In this new activity, the student must use two emotions: tired (a version of sadness) and excited (a version of happiness). The child must identify, from a set of images of situations, the one in which the characters show one of these emotions, according to what the game audio asks them to do.

#### Session 6

Once again, the session takes place at the zoo.

**Activity 1:** In this new activity, students will use two emotions: tired (a version of sadness) and excited (a version of happiness). From a face with missing eyes, students must select and drag the eyes that best describe the emotion expressed by the mouth (tired or excited).

**Activity 2:** For this activity, the children must perform two actions:

1. The student must select the friend who represents the emotion that the game is asking for.
2. They must listen and memorize the relationship between the color of the plate of food or water and the animal. To do this, a large animal will appear in the middle of the screen and two plates of food of different colors will appear below, which will be used as buttons. The animal in the middle will change, but the food plates will not. The child must press the button for the food or water plate that corresponds to each of the animals (e.g., lion, red plate-button; giraffe, blue plate-button).

**Activity 3:** Different situations will appear on the screen, in which one of the children has no face. The student must select which face the child receiving the action would be making (e.g., one child teasing another with a spider, a child excluded from a group of friends, among others.).

**Activity 4:** For this activity, the children must perform two actions:

1. The student must select the friend who represents the emotion that the game is asking for.
2. They must listen and memorize the relationship between the shower faucet color and the animal. To do this, a large animal will appear in the middle of the screen and two shower faucets of different colors will appear below, which will be used as buttons. The animal in the middle will change, but the faucets will not. The child must press the shower faucet button that corresponds to each animal (e.g., bear, black shower faucet button; giraffe, blue shower faucet button).

#### Session 7

This session will take place in the setting of a house's backyard.

**Activity 1:** Several faces will appear on screens, continuously changing emotions. The student must touch the faces only when they are expressing the indicated emotion and not touch them when they have another expression.

**Activity 2:** In this exercise, the child will play with two new emotions: frustrated and proud. They must select from a set of situations those in which the characters represent the requested emotion.

**Activity 3:** A large face will appear on the screen, continuously changing emotions. Students should touch the large face when it shows one of the indicated emotions and not touch it when it shows other emotions. The emotions that will be shown are: happy, sad, scared/fearful, and angry.

**Activity 4:** Various situations will appear in which one of the children has no face. The student must select which face the child receiving the action would be making.

#### Session 8

Once again, this session will take place in a classroom setting.

**Activity 1:** Students should do the following in this exercise:

1. Listen and observe one of the strategies they can use to calm their frustration:

- Breathe through your nose, counting mentally to 3
- Hold your breath while counting to 3 in your head
- Exhale while counting to 3 in your head.

2. Next, the student should put the strategy described above into practice and simulate a child's breathing. To do this, they will need to do the following:

- First, they must press and hold the button on the child's nose for three seconds, that is, until the red light on the traffic light on the left side of the screen turns on. At that point, they must release the button.
- Then they must press and hold the button on the child's chest for three seconds, until the red light on the traffic light on the left side of the screen turns on. At that point, they should release the button.
- Then you must hold down the button on the child's mouth for three seconds, that is, until the red light on the traffic light on the left side of the screen turns on. At that point, you must stop pressing the button.
  3. Finally, you will complete the entire cycle. You must hold down the button on the nose, then on the chest, and finally on the mouth. For each of these cycles, you must hold the button down for three seconds, that is, until the red light on the traffic light on the left side of the screen turns on. Then you should stop pressing the button.

**Activity 2:** A large face will appear in the middle of the screen and two small faces below that will be used as buttons (one calm and one angry). The face in the middle will change, but the button faces will not. Each time the large face shows an emotion (calm or angry), the child should touch the opposite emotion (e.g., if the large face is calm, they should touch the button with the angry face).

**Activity 3:** In this activity, they will practice the calming method again. To do this, they will simulate a child's breathing by doing the following:

1. First, they must hold down the button on the child's nose for three seconds, that is, until the red light on the traffic light on the left side of the screen turns on. Then they must stop pressing the button.
2. Then you will have to hold down the button on the child's chest for three seconds, that is, until the red light on the traffic light on the left side of the screen turns on. Then you should stop pressing the button.
3. Then you must hold down the button on the child's mouth for three seconds, that is, until the red light on the traffic light on the left side of the screen turns on. At that point, you must stop pressing the button.
4. Finally, you will complete the entire cycle. You must hold down the button on the nose, then on the chest, and finally on the mouth. For each of these cycles, you must hold the button down for three seconds, that is, until the red light on the traffic light on the left side of the screen turns on. Then you should stop pressing the button.

**Activity 4:** A large animal face will appear in the middle of the screen and two small animal faces below it will be used as buttons (one calm and one angry). The face in the middle will change, but the button faces will not. Each time the large face shows an emotion, the child should touch the opposite emotion (e.g., if the large lion face is calm, they should touch the button with the angry monkey face). Small animal faces will also appear around the screen, changing emotions, which they should not touch.

#### Session 9

**Activity 1:** The ferret appears and asks the avatar to observe different situations and identify problems. For each trial, four situations are presented (three neutral or without conflict and one with a problem). The child will have to choose which situation is problematic. Then, they will be asked to identify the type of emotion that the problem generates in the characters.

**Activity 2:** In this exercise, the children must listen to the sounds of the animals, memorizing the order in which they heard them. Then, they must drag the image of the animal into the boxes in the same order in which they heard them.

**Activity 3:** Students are presented with different situations, and for each one, they must select the problem. Once they select the problem, they must select the emotion they would feel if that situation happened to them.

**Activity 4:** In this game, the characters arrive at the beach. The student must observe and memorize the order in which the beach objects light up (flash), and then drag them and place them in the boxes in the order in which they lit up.

#### Session 10

**Activity 1:** In this activity, the children must play at packing their backpack to go to school. To do this, they must listen to the objects that are named (pencil, eraser, scissors, ruler, juice, among others.), memorizing the order in which they were named, and then drag the images of the objects to the boxes in the memorized order.

**Activity 2:** In this next exercise, children must look at a set of images. These images represent different moments in the same story that depicts a problem. Students must order the images by dragging them to the boxes in the correct sequence of the story, following the chronological order.

**Activity 3:** Children must observe and memorize the order in which the boxes in the "luche" game light up. They must then touch the boxes in the same order.

**Activity 4:** Children should look at a set of images. These images represent different moments in the same story that depicts a problem. Students should arrange the images by dragging them into the boxes in the correct sequence of the story, following the chronological order.

#### Session 11

**Activity 1:** Children must put the sequence in order according to the breathing method for calming down. To do this, students must observe and put the five images that appear at the bottom of the tablet in a logical sequence, using the method they learned for calming down when faced with a problem. The sequence will be as follows: problem, cross arms, breathe in through the nose, breathe out through the mouth, and identify the solution.

**Activity 2:** In this activity, children must play at packing their backpack to go to school. To do this, they must listen to the objects that are named (pencil, eraser, scissors, ruler, juice, among others), memorizing the order in which they were named, and then drag the images of the objects to the boxes in the memorized order.

**Activity 3:** Each rehearsal will present different problem situations. Students will order the sequence according to the breathing method to calm down when faced with a problem. The sequence will be as follows: problem, cross arms, breathe in through the nose, breathe out through the mouth, and identify the solution.

**Activity 4:** Students must listen and memorize the order in which the game audio mentions the colors of the circles. They must then touch them in the same order in which they were mentioned.

#### Session 12

**Activity 1:** Children will listen to different ingredients for making a cake (flour, sugar, milk, etc.) in a specific order. They must memorize the order in which they were named and then drag them (keeping the order) into the boxes.

**Activity 2:** This time, the setting is a beach. Several problematic situations will be presented that the student must solve by selecting the solution.

**Activity 3:** Children are shown a piano, where the keys light up in a specific order. Children must first observe and memorize that order and then play the keys in the same order.

**Activity 4:** A situation is shown on the screen. The children must perform two actions:

1. Select a ticket if the situation is a good idea or press the X if it is a bad idea.
2. Choose a different way of acting in that situation that will make everyone involved happy.

#### Session 13

**Activity 1:** Children will be presented with various problematic situations and asked to choose the solution that worked best and made everyone happiest. They will be given a choice between negative and positive solutions.

**Activity 2:** The children will play a game of putting fruit into a bag. To do this, they will have to listen and memorize the order in which the fruits are mentioned. Then they will have to drag them to the white boxes in the same order.

**Activity 3:** Children will play a puzzle game to discover how the different problematic situations presented end.

**Activity 4:** Several children will appear on the screen in a horizontal row. The student must first observe the exercises the children are doing and memorize the order in which they were performed, then touch each exercise in the memorized order.

#### Session 14

**Activity 1:** Children should observe and memorize the order in which the pieces of a puzzle light up. Then they will have to touch them in the same order in which they lit up.

**Activity 2:** In this activity, children will discover what happens when they come up with a good solution. To do this, they must select three possible solutions to a problem.

**Activity 3:** Children should listen to and memorize the order in which the audio mentions the masks of different characters (pirate, vampire, ghost, among others). Then they should touch the masks in the same order in which they were mentioned.

**Activity 4:** Children will play a puzzle game to discover how to solve different problems presented.

#### Session 15:

**Activity 1:** Based on different solutions (good and bad) to problematic situations, students must identify how the characters feel and then select whether the solution was good (by selecting the ticket) or bad (by selecting the X).

**Activity 2:** Children should observe and memorize the order in which the pieces of puzzles that form different insects light up. Then they will have to touch them in the same order in which they lit up.

**Activity 3:** Based on different solutions (good and bad) to problematic situations, students must identify how the characters feel and then select whether the solution was good (by selecting the ticket) or bad (by selecting the X).

**Activity 4:** Children should play a game of "putting away" toys (kites, stuffed animals, cars, etc.). To do this, they will have to listen and memorize the order in which the audio mentions the toys. Then they must drag the toys to the white boxes in the same order in which they were mentioned.

#### Session 16:

**Activity 1:** Children must observe and remember the order in which the balls light up (flash). They must then touch them in the same order.

**Activity 2:** In this activity, based on different solutions (good or bad), children must identify the emotion felt by the characters in the problem. Then they will have to identify whether the solution was good (by touching the ticket) or bad (by touching the X).

**Activity 3:** Students must listen and memorize the order in which the means of transportation are mentioned. Then they must touch them in the same order.

**Activity 4:** In this activity, based on different solutions (good or bad), children must identify the emotion felt by the characters in the problem. Then they will have to identify whether the solution was good (by touching the ticket) or bad (by touching the X).

#### Session 17:

**Activity 1:** Children will play "dress up" as different characters. They will be told different characters that the child must dress up as (explorer, astronaut, angel, etc.). The student has to think about what they will need to look like the character. A total of 9 options will be shown from which they must choose, 3 of which will be the correct ones that they must mark.

**Activity 2:** The activity begins with a memory game where children discover that there are three ways to know how a person feels: listening, observing, and asking questions. After this, the children will practice their observation skills. To do this, different scenes will appear where the main character will not have a face, and the students will have to identify the character's emotion.

**Activity 3:** Different activities will be described, and the children will have to think about what they will need to do them (drawing, cutting, building, etc.). A total of nine options will be shown, of which three will be the correct ones to mark.

**Activity 4:** The activity begins with a map on the screen where children must find the 3 ways to know how a person feels. To listen, they will have to select a concert; to observe, they will have to select an art museum; and to ask, they will have to select a school. After that, they will have to identify two things that can make different people happy. For example: What could make a child your age happy? What could make a mother happy? Etc.

#### Session 18:

**Activity 1:** The activity begins with three cards on the screen, which the child must touch to turn over and remember the three ways to know how a person feels. In this activity, they will practice the skill of asking questions. To do this, they will observe various situations: the character will not have a face or make any sounds, so the children must first guess the characters' emotions and then play at asking them how they feel.

**Activity 2:** Different activities will be described, and the children will have to think about what they will need to do them (draw, cut, build, etc.). A total of 9 options will be shown, of which 4 will be the correct ones to mark.

**Activity 3:** In this activity, children must identify two things that can make different people angry. For example: What could make a child your age angry? What could make a mother angry? Etc.

**Activity 4:** Children will play "dress up" as different characters. They will be told different characters that the child must dress up as (explorer, astronaut, angel, etc.). The student has to think about what they will need to look like the character. A total of 9 options will be shown from which to choose, 4 of which will be the correct ones to mark.

#### Session 19

**Activity 1:** Different household activities will be mentioned (washing dishes, sweeping, mopping, etc.), and the child will have to think about what they will need to do them. A total of 9 options will be shown, of which 3 will be the correct ones to mark.

**Activity 2:** Children practice different ways of finding out how a person feels: listening, observing, and asking questions. To do this, different situations will be presented on the screen in one of the following formats:

1. The main character has no face and does not make any sounds (asking).
2. The main character has no face but makes a sound related to their emotion (listen)
3. The main character has a face but does not make any sound (observe)

The child must select the option that allows them to know how the character feels.

**Activity 3:** Different places where the ferret will go for a walk and some related activities will be mentioned (e.g., building a sandcastle on the beach, having a picnic in the park, etc.). The child has to think about what they will need to do this. A total of nine options will be shown, of which three will be the correct ones to mark.

**Activity 4:** In this activity, children must identify two things that can make different people sad. For example: What could make a child your age sad? What could make a mother sad?, among others.

#### Session 20

**Activity 1:** In this activity, children will learn that the same person can feel different ways. To do this, they must select the things they think could make the same character feel two different emotions.

**Activity 2:** Different household chores will be mentioned (washing dishes, sweeping, mopping, etc.), and the child has to think about what they will need to do them. A total of nine options will be shown, four of which will be correct and must be marked.

**Activity 3:** In this game, children must listen to the "mysterious voice." Then they have to select how the character feels and who they think is saying the phrase (e.g., mom, firefighter, child their age, elderly person, etc.).

**Activity 4:** Different places where the ferret will go for a walk and some associated activities will be mentioned (e.g., building a sandcastle on the beach, having a picnic in the park, etc.). The child has to think about what they will need to do this. A total of 9 options will be shown, of which 4 will be the correct ones to mark.

#### Session 21

**Activity 1:** Different places where the ferret will go for a walk and some associated activities will be mentioned (e.g., building a sandcastle on the beach, having a picnic in the park, etc.). The child has to think about what things they will need to do this. A total of 9 options will be shown, of which 3 will be the correct ones to mark.

**Activity 2:** In this activity, children will learn that different people can feel the same way. The exercise will take place in an amusement park setting. For each attraction in the park, the child must select the two characters in the scene who are feeling the same emotion.

**Activity 3:** Children will be invited to play different sports, for which they will have to dress in professional sportswear (cyclist, swimmer, tennis player, etc.). Children will have to think about what they need to achieve this. A total of 9 options will be shown, of which 3 will be the correct ones to mark.

**Activity 4:** In this activity, children must identify two things that might scare different people. For example: What might scare a child your age? What might scare a mother? Etc.

#### Session 22

**Activity 1:** In this activity, children will learn that different people can feel differently about the same thing. To do this, they will have to observe different scenes featuring two faceless characters. The child will have to identify the emotion of each of the characters.

**Activity 2:** Different places where the ferret will go for a walk and some associated activities will be mentioned (e.g., building a sandcastle on the beach, having a picnic in the park, etc.). The child has to think about what things they will need to do this. A total of nine options will be shown, of which four will be the correct ones to mark.

**Activity 3:** In this activity, children will learn what could cause a person to switch from one emotion to another. They will observe different children expressing emotions and must select the option that would allow the child to change their emotion, according to the audio instructions (e.g., select the option that causes a child to switch from angry to calm).

**Activity 4:** Children will be invited to play different sports, for which they will have to dress in professional sportswear (cyclist, swimmer, tennis player, etc.). Children will have to think about what they need to achieve this. A total of 9 options will be shown, of which 4 will be the correct ones to mark.

**Session 23**

**Activity 1:** Children will be invited to perform different jobs or tasks, for which they will have to think about what they need to be able to do those jobs (gardener, teacher, carpenter, etc.). A total of 9 options will be shown, of which 3 will be the correct ones to mark.

**Activity 2:** In this activity, children will learn that what we do affects others and causes their emotions to change. To do this, they will observe different scenes where one character performs an action on another. Then they will have to identify how the character receiving the action feels.

**Activity 3:** Children will be invited to perform different jobs or tasks, for which they will have to think about what they need to be able to do those jobs (judge, dentist, doctor, etc.). A total of 9 options will be shown, of which 3 will be the correct ones to mark.

**Activity 4:** In this activity, children must identify two things that can cause anger in different people. For example: What could make a child your age angry? What could make a mother angry? Etc.

#### Session 24

**Activity 1:** In this activity, children will learn that what we do affects others and causes their emotions to change. To do this, they will observe different scenes where one character performs an action on another. Then they will have to identify how the character receiving the action feels.

**Activity 2:** Children will be invited to perform different jobs or tasks, for which they will have to think about what they need to be able to do those jobs (judge, dentist, doctor, etc.). A total of 9 options will be shown, of which 4 will be the correct ones to mark.

**Activity 3:** In this activity, children will learn what they can do to help a person move from one emotion to another. They will observe different situations in which the main character feels a negative emotion and select what they can do to help them move to a positive emotion.

**Activity 4:** Children will be invited to perform different jobs or occupations, for which they will have to think about what they need to be able to perform those jobs (judge, dentist, doctor, etc.). A total of 9 options will be shown, of which 4 will be the correct ones to mark.
