# Supplementary material for: Japi 2.0, a gaming platform to stimulate cognitive and socio-emotional skills in early childhood: results of a pilot randomized controlled trial
Source: Child Adolesc Psychiatry Ment Health. 2026 Jul 21;20:145. doi: 10.1186/s13034-026-01133-1 (PMC13673852; doi:10.1186/s13034-026-01133-1)
Supplement: Supplementary file 2 [file 13034_2026_1133_MOESM2_ESM.docx]

**Suppl. 2. Images from the Japi 2.0 video game.**

The following images correspond to different activities in the Japi video game:

At the start of the game, each child chooses their avatar. They can choose gender, hair color, skin color, and clothes.

This activity corresponds to the emotion recognition skill. The student must identify how the girl who pushes the other girl off the swing feels.

This activity corresponds to the inhibitory control skill. Several items of different colors will appear on the screen. The child should touch the items only when they turn the color requested by the game (e.g., touch the toy boats when they turn orange).

This activity corresponds to the planning skill. Children will play "dress up" as different characters. They will be told different characters that the child must dress up as (explorer, astronaut, angel, etc.). The student has to think about what they will need to look like the character

This game corresponds to the empathy skill. Children must discover how different characters feel either, observing their face or listening to the "mysterious voice. If none of these alternatives are available, children must ask directly to the character: “How do you feel?”. With this activity children will discover the three ways to know how someone is feeling.
